# Grape-Seed Proanthocyanidins are Able to Reverse Intestinal Dysfunction and Metabolic Endotoxemia Induced by a Cafeteria Diet in Wistar Rats

**DOI:** 10.3390/nu11050979

**Published:** 2019-04-29

**Authors:** Carlos González-Quilen, Katherine Gil-Cardoso, Iris Ginés, Raúl Beltrán-Debón, Montserrat Pinent, Anna Ardévol, Ximena Terra, M. Teresa Blay

**Affiliations:** MoBioFood Research Group, Departament de Bioquímica i Biotecnologia, Universitat Rovira i Virgili, 43007 Tarragona, Spain; carlosalberto.gonzalez@urv.cat (C.G.-Q.); katherine.gil@urv.cat (K.G.-C.); iris.gines@urv.cat (I.G.); raul.beltran@urv.cat (R.B.-D.); montserrat.pinent@urv.cat (M.P.); anna.ardevol@urv.cat (A.A.); mteresa.blay@urv.cat (M.T.B.)

**Keywords:** flavan-3-ol, gut, inflammation, obesity, tight junction, Ussing chamber, transepithelial electrical resistance

## Abstract

We evaluated the effectiveness of pharmacological doses of grape-seed proanthocyanidin extract (GSPE) in reversing intestinal barrier alterations and local inflammation in female Wistar rats fed a long-term obesogenic diet. Animals were fed a 17-week cafeteria diet (CAF diet), supplemented with daily GSPE doses (100 or 500 mg kg^−1^ body weight) during the final two weeks. CAF diet enhanced the intestinal permeation of an orally administered marker (ovalbumin, OVA) and increased the plasma levels of tumor necrosis factor-α (TNF-α) and lipopolysaccharides (LPS) in 2–3-fold. Ex vivo Ussing chamber assays showed a 55–70% reduction in transepithelial electrical resistance (TEER) and increased the TNF-α secretions in both small and large intestinal sections with a 25-fold increment in the ileum. Ileal tissues also presented a 4-fold increase of myeloperoxidase (MPO) activity. Both GSPE-treatments were able to restitute TEER values in the ileum and colon and to reduce plasma LPS to basal levels without a dose-dependent effect. However, effects on the OVA permeation and TNF-α secretion were dose and section-specific. GSPE also reduced ileal MPO activity and upregulated claudin 1 gene expression. This study provides evidence of the efficacy of GSPE-supplementation ameliorating diet-induced intestinal dysfunction and metabolic endotoxemia when administered at the end of a long-term obesogenic diet.

## 1. Introduction

It has been reported that there is a causal relationship between pathophysiological changes in the intestinal epithelium and obesity in animal models and humans [1,2,3]. Studies aiming to identify natural compounds that modulate intestinal alterations in obesity are therefore promising future therapeutic strategies [2,4].

The rising prevalence of obesity in Western societies and developing countries is a serious public health concern that, in part, results from the consumption of unbalanced hypercaloric diets [5,6]. High-saturated fat/high refined-carbohydrate diets, that are low in fiber and plant flavonoids, induce not only weight gain in humans and laboratory animals, but also alterations in gut microbiota (dysbiosis) [7,8,9] and intestinal dysfunction (increased permeability and local inflammation) [10].

Increased intestinal permeability leads to a higher flow of intestinal endotoxins, e.g., lipopolysaccharides (LPS) deriving from luminal gram-negative bacteria, to the inner intestinal layers [11,12,13]. This causes a local inflammatory response in the intestine that aggravates barrier function deficiency in a vicious cycle. When LPS reaches the general circulation due to the inability of the intestinal immune response to neutralize it, it can spread systemically causing metabolic endotoxemia and obesity-related complications such as adipose tissue dysfunction as well as systemic low-grade inflammation [14].

Intestinal permeability and inflammatory response are site-specific processes, i.e., the small and large intestines behave in different ways in terms of permeability increase and susceptibility to inflammation [15,16]. The duration of the dietary feeding period has a strong influence on the initiation of intestinal dysfunction and obesity in animals consuming a high-fat/high-sugar diet. Rats fed an obesogenic diet show evidence of intestinal dysfunction at 8–12 weeks [3,8,17,18]. By this time, an increment of permeability (most commonly measured as plasma concentration of an orally administered marker), proinflammatory cytokine production (TNF-α, IL-6, etc.) and myeloperoxidase (MPO) activity are evidenced in the intestinal mucosa. The downregulation of tight-junction (TJ)-related genes and proteins (zonula occludens 1, occludin and claudin 1) also occurs.

Anti-obesity treatments have proven to be of limited effectiveness. Complementary dietary strategies such as bioactive compounds with anti-obesity effects could, therefore, be an adjunctive support to current therapies and reinforce obesity treatments. Indeed, the supplementation of hypercaloric diets with flavonoid sources could become a reliable strategy for correcting the intestinal dysfunction associated with the obesogenic process [18,19]. Notably, in European countries, the average habitual intake of flavonoids is considerably below the amounts used in most dietary intervention studies [20].

Multiple studies demonstrate the beneficial effects of a grape-seed proanthocyanidin extract (GSPE) on obesity-related outcomes, including the amelioration of intestinal dysfunction [19,21,22,23]. Proanthocyanidins are oligomeric and polymeric flavan-3-ols, mainly constituted of catechin and epicatechin subunits. They are an important component of plant-based diets and are abundant in green tea, certain fruits and beverages and in spices such as cinnamon [24].

We have described a combination of GSPE doses and frequencies of administration that can effectively prevent some of the metabolic and intestinal alterations associated with diet-induced obesity in rats [18,23]. In a previous study, we also tested the effect of nutritional doses of the GSPE (5–50 mg kg^−1^ d^−1^) after 15 weeks of cafeteria (CAF) diet administration. We evidenced a reduction of oxidative stress (reactive oxygen species and iNOS mRNA expression) and inflammatory markers (MPO activity and IL-1β mRNA expression) in ileal tissues, as well as the upregulation of zonula occludens 1 gene expression. Nevertheless, we found no differences in plasma LPS levels between experimental groups at these doses. Our hypothesis is that, in rats fed a CAF diet for a period of 17 weeks leading to obesity, supplementation with the GSPE at pharmacological doses during the final two weeks can reverse the diet-associated intestinal dysfunction and metabolic endotoxemia.

In this study, we determined the effectiveness of two pharmacological doses of GSPE (100 and 500 mg kg^−1^ body weight), administered daily for two weeks at the end of a 17-week obesogenic diet. The doses and conditions of administration of the GSPE were evaluated by in vivo and ex vivo measurements in both small (duodenum and ileum) and large (colon) intestines. We focused on intestinal permeability, local inflammatory status, metabolic endotoxemia and systemic inflammation. Here, we corroborate the relationship between CAF diet consumption, metabolic endotoxemia and the associated intestinal dysfunction. Additionally, we provide evidence of the counteractive effect of GSPE on the alterations induced by a long-term CAF diet (obese animals) at the intestinal and systemic levels.

## 2. Materials and Method

### 2.1. Grape-Seed Proanthocyanidin Extract

The grape-seed proanthocyanidin extract (GSPE) was provided by Les Dérivés Résiniques et Terpéniques (Dax, France). The GSPE administered in this study (batch number 124029) has the following composition: monomers of flavan-3-ols (21.3%), dimers (17.4%), trimers (16.3%), tetramers (13.3%) and oligomers (5–13 units; 31.7%) of proanthocyanidins. A detailed analysis of the monomeric, dimeric, and trimeric structures can be found in the work by Margalef et al. [25].

### 2.2. Experimental Animals

Forty-seven-week-old female Wistar rats (240–270 g) purchased from Charles River Laboratories (Barcelona, Spain) were individually caged in animal quarters at 22 °C with a 12-h light/12-h dark cycle and fed a standard chow diet (STD diet; Panlab 04, Barcelona, Spain) and tap water *ad libitum*. After an acclimation period, animals were randomly distributed into four experimental groups (*n* = 10). The control group (STD group) received only the STD diet. The other groups were fed a CAF diet as a model of high saturated-fat/high refined-carbohydrate diet until the end of the animal experiment. The CAF diet was offered ad libitum and replenished every day with a quantity that was enough for 17 weeks. CAF-fed animals also had free access to standard chow. The composition of the diets supplied is shown in Table 1.

**Figure 1:**
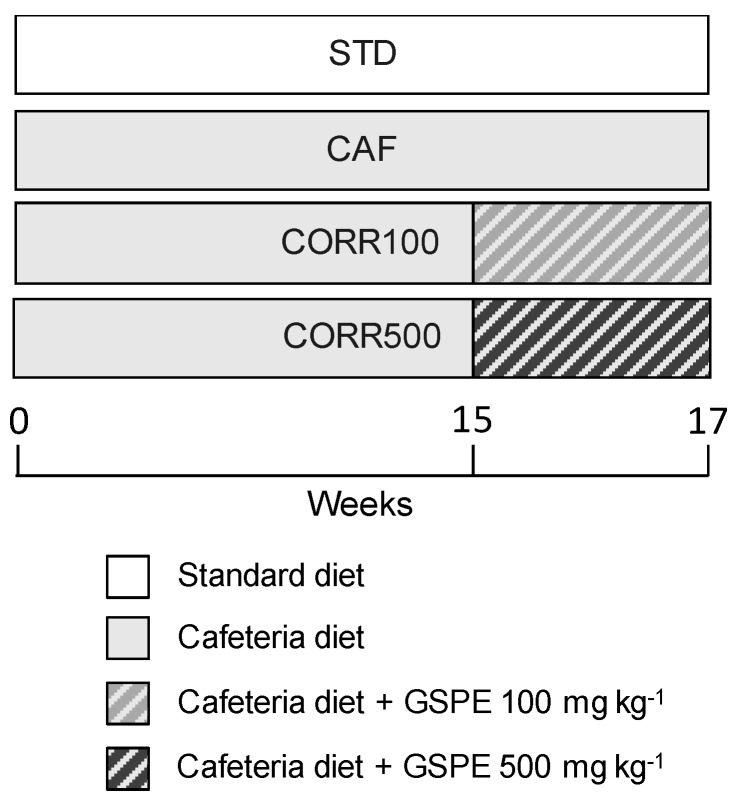
The schematic diagram of the experimental design. STD group, rats fed standard chow diet; CAF group, rats fed cafeteria (CAF) diet; CORR100 group, CAF-fed rats supplemented with 100 mg kg^−1^ body weight of grape-seed proanthocyanidin extract (GSPE); CORR500 group, CAF-fed rats supplemented with 500 mg kg^−1^ bw of GSPE. GSPE doses were administered daily by oral gavage during the final two weeks of the animal experiment. The number of animals included in this study was *n* = 10 for each group.

During the final two weeks of the CAF intervention, two CAF-diet groups received daily GSPE doses of 100 and 500 mg kg^−1^ bw as a corrective treatment (groups CORR100 and CORR500, respectively). The GSPE was dissolved in water and orally gavaged every day to each animal at 18:00 h in a final volume of 0.5 mL. Non-supplemented animals received water as a vehicle. The experimental design is shown in Figure 1. All procedures involving the care and use of animals in this work were reviewed and approved by The Animals Ethics Committee of the Universitat Rovira i Virgili (code: 0152S/4655/2015).

### 2.3. Blood and Tissue Collection

At the end of the study, the animals were fasted for 14 h, anesthetized with sodic pentobarbital (70 mg kg^−1^ bw; Fagron Iberica, Barcelona, Spain) and exsanguinated from the abdominal aorta. The total blood was collected using heparin (Deltalab, Barcelona, Spain) as an anticoagulant. Plasma was obtained by centrifugation (1500 × g for 15 min at 4 °C) and stored at −80 °C until analysis. White adipose tissue depots were rapidly removed and weighed. The small intestine, defined as the portion of the gastrointestinal tract between the pylorus and the ileocecal valve, was dissected. Colon tissues were also removed. Five-centimeter segments of the duodenum, ileum and colon were taken for Ussing chamber assays. Leftover segments of small and large intestines were stored at −80 °C until analysis.

### 2.4. Morphometric and Biochemical Parameters

Body weight was monitored weekly until the end of the experiment. Body weight gain was calculated by subtracting the initial body weight from the final body weight. Adiposity was expressed as an adiposity index, which was based on total fat pad measurements. This was computed for each rat as previously described [26]. These variables were evaluated as physiological indicators of the degree of obesity in the experimental animals.

Enzymatic colorimetric kits were used to measure the plasma levels of glucose (QCA, Amposta, Spain) and triacylglycerols (TAG) (QCA, Amposta, Spain).

### 2.5. LPS and TNF-α Plasma Determinations

Plasma LPS levels were determined using a Pyrochrome lysate mix diluted in Glucashield buffer (Associates of Cape Cod, E. Falmouth, MA, USA), which inhibits cross-reactivity with (1→3)-β-d-glucans. Plasma concentrations of tumor necrosis factor-α (TNF-α) were measured by ELISA (Merck Millipore, Madrid, Spain) with a sensitivity of 4.2 pg mL^−1^.

### 2.6. Oral Intestinal Permeability Test

In vivo intestinal permeability was assessed using the ovalbumin (OVA) test at 17 weeks, for which the animals were previously fasted for four hours. OVA (Sigma-Aldrich, Madrid, Spain) was administered by oral gavage at 250 mg kg^−1^ bw in a final volume of 0.5 mL of phosphate buffer solution. One hour later, blood was collected from the saphenous vein, then heparinized and centrifuged (12,000 × g for 10 min at 4 °C). Plasma OVA levels were determined by ELISA (MyBioSource, Madrid, Spain) with a detection range of 16–10,000 pg mL^−1^.

### 2.7. Ex Vivo Assessment of Intestinal Dysfunction: Intestinal Barrier Integrity and Local Inflammation

Ex vivo intestinal permeability and local inflammation were evaluated in an Ussing chamber system (Dipl.-Ing. Muβler Scientific Instruments, Aachen, Germany) by measuring transepithelial electrical resistance (TEER) and TNF-α secretions in the basolateral medium. At the end of the experiment, fresh intestinal tissues (duodenum, ileum and colon) were immediately placed in a cold oxygenated Krebs-Ringer bicarbonate buffer (KRB buffer), dissected to remove serosal and muscular layers, and placed on 0.237 cm^2^ aperture Ussing chambers. Mucosal preparations were mounted within 10 min following euthanasia and apically and basolaterally bathed with 1.5 mL of the KRB buffer. The basolateral bathing solution, which contained 10 mM of glucose (Panreac, Barcelona, Spain), was osmotically balanced on the apical compartment with 10 mM of mannitol (Sigma, Madrid, Spain). Bathing solutions were oxygenated and circulated in water-jacketed reservoirs maintained at 37 °C. The transepithelial potential difference (PD) was short-circuited through Ag-AgCl electrodes with a voltage clamp that corrected for fluid resistance. TEER (ohm.cm^2^) was calculated from the transepithelial PD and short-circuit current in accordance with Ohm’s law.

After a 20-min equilibration period, the KRB buffer in the apical and basolateral compartments was replaced by a fresh KRB buffer containing 10 mM of glucose and protease inhibitors (10 µM amastatin (Enzo Life Sciences, Madrid, Spain), 500 KIU aprotinin (Sigma, Madrid, Spain) and 0.1 % bovine serum albumin (BSA) fatty acid free). Mucosal preparations were incubated for an additional 30 min, after which basolateral media were collected for determining TNF-α secretions by ELISA (Merck Millipore, Madrid, Spain).

### 2.8. Quantification of MPO Activity in the Ileum

MPO activity was used as an indicator of neutrophil accumulation in the rat ileum. Tissue samples were homogenized with a TissueLyser LT system (Qiagen, Hilden, Germany) in 50 mM of potassium phosphate buffer (Panreac, Barcelona, Spain). The homogenate was centrifuged at 15,000 × g for 15 min at 4 °C, and the resulting supernatant was discarded. The pellet was then homogenized with hexadecyltrimethylammonium bromide (HTBA) (Sigma-Aldrich, Madrid, Spain) and 50 mM of potassium phosphate buffer. The homogenate was sonicated (20 s), subjected to three freeze-thaw cycles, and centrifuged at 15,000 × g for 10 min at 4 °C. To determine MPO activity we used an adaptation of the Lenoir method [27]. The supernatant was mixed into a solution of a phosphate buffer, 0.22% guaiacol (Sigma-Aldrich, Madrid, Spain) and 0.3% H_2_O_2_ (Sigma-Aldrich, Madrid, Spain), and the absorbance was read at 470 nm. MPO activity was expressed as U per mg of protein in the final fraction.

### 2.9. Tissue RNA Extraction and Gene Expression Analysis by RT-qPCR

The total RNA was extracted from 50 mg of ileum using TRIzol reagent (Thermo Fisher Scientific, Waltham, MA, USA) in accordance with the manufacturer’s instructions. Complementary DNA (cDNA) was obtained from 1 µg of mRNA using the High Capacity cDNA Reverse Transcription kit (Applied Biosystems, Madrid, Spain) following the manufacturer’s instructions. Quantitative polymerase chain reaction (qPCR) amplification and detection were performed in a qPCR system (Applied Biosystems, Madrid, Spain) using the TaqMan Universal PCR Master Mix (Applied Biosystems, Madrid, Spain) and the respective specific TaqMan probes (Applied Biosystems, Madrid, Spain): Rn02116071_s1 for zonula occludens 1 (*Tjp1*), Rn00587389_m1 for junctional adhesion molecule 1 (*F11r*), Rn00581740_m1 for claudin 1 (*Cldn1*), Rn02063575_s1 for claudin 2 (*Cldn2*). The results were normalized with respect to the cyclophilin A gene (*Ppia*) (Rn00690933_m1). Reactions were performed using the following thermal profile: 2 min at 50 °C, 2 min at 95 °C, and 40 cycles of 15 s at 95 °C and 2 min at 60 °C. The relative mRNA expression levels were calculated following the 2^−ΔΔCt^ method [28], where ΔCt=Ct gene of interest−Ct cyclophilin and ΔΔCt=ΔCt treated samples−the mean of ΔCt control samples.

### 2.10. Statistical Analysis

Results are expressed as the mean value ± the standard error of the mean (SEM). Statistical comparisons between groups were assessed by a two-sided Student’s *t*-test or ANOVA followed by Tukey’s HSD test when the variances were equal and Dunnett’s T3 test when they were not. *P*-values < 0.05 were considered statistically significant. Analyses were performed with IBM SPSS statistics 22 software (SPSS Inc., Chicago, IL, USA).

## 3. Results

### 3.1. Morphometric and Biochemical Parameters

Here, we tested the GSPE supplementation (100 and 500 mg kg^−1^ bw per day) in Wistar rats during the final two weeks of a 17-week CAF diet.

Morphometric and biochemical parameters are presented in Table 2. To assess the influence of the GSPE treatment on the CAF diet detrimental effects, we first tested its effectiveness in controlling body weight and adiposity. GSPE-supplementation did not bring about any change in the final body weight or adiposity index compared to the CAF group, even though the body weight gain of the CORR500 group showed a statistically significant reduction. Regarding biochemical parameters, GSPE-supplementation did not produce any significant changes in the glucose or triacylglycerol levels. Non-statistically significant differences were found between the CORR100 and CORR500 groups for the parameters already mentioned.

### 3.2. In Vivo Intestinal Permeability

**Figure 2:**
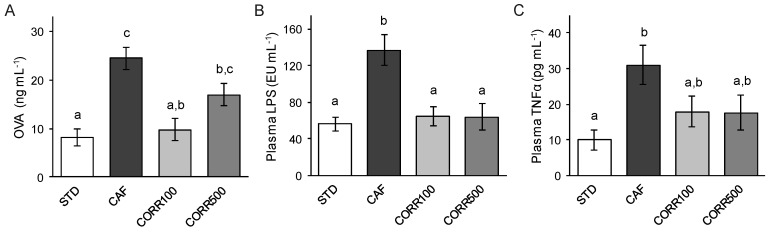
The effect of the grape-seed proanthocyanidin extract (GSPE) treatment on intestinal permeability, metabolic endotoxemia and systemic inflammation in diet-induced obese rats. Values represent mean ± SEM; *n* = 8–10. Different letters indicate the statistically significant differences between groups (*p* < 0.05); ANOVA test followed by Tukey’s HSD test when variances were equal and Dunnett’s T3 test when they were not.

The effects of the CAF diet and GSPE on intestinal permeability were determined in vivo by the measurement of OVA concentrations in plasma, 1 h after oral administration. After 17 weeks, the CAF group showed a 3-fold increase of plasma OVA levels compared to the STD group (24.2 ± 2.2 and 8.2 ± 1.7 ng mL^−1^ respectively; Figure 2A). Interestingly, GSPE supplementation decreased the OVA permeation by 30–60% with respect to the CAF group, but only the CORR100 group showed a significant reduction in this parameter (9.8 ± 2.3 ng mL^−1^).

### 3.3. Metabolic Endotoxemia and Systemic Inflammation

The expected increased influx of the endotoxin through the intestinal epithelium of obese animals and the potential ameliorative effect of GSPE on metabolic endotoxemia were assessed by determining plasma LPS levels. The CAF group showed significantly higher LPS levels in plasma with respect to the STD group (136.9 ± 17.1 and 56.2 ± 7.7 EU mL^−1^ respectively; Figure 2B). Plasma LPS levels estimated in GSPE-treated groups were comparable to the STD group (64.7 ± 10.8 and 64.1 ± 14.8 EU mL^−1^ for CORR100 and CORR500 respectively), which represents a significant reduction with respect to the CAF group without a dose-dependent effect.

TNF-α was measured in plasma as a systemic inflammation marker. The CAF group showed a 3-fold increase in plasma TNF-α with respect to the STD group (30.9 ± 7.7 and 10 ± 2.8 pg mL^−1^). A non-statistically significant reduction in this marker was found in the CORR100 and CORR500 groups compared to the CAF group (17.9 ± 4.4 and 17.6 ± 4.9 pg mL^−1^ respectively; Figure 2C).

### 3.4. Ex Vivo Intestinal Barrier Integrity

**Figure 3:**
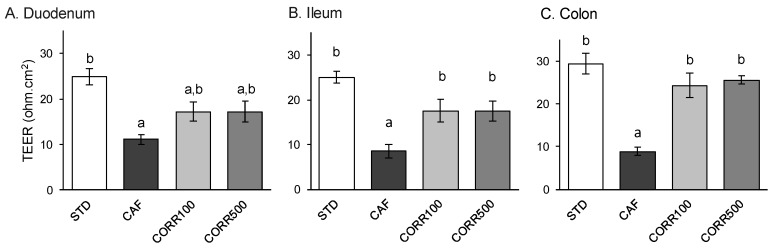
The effect of the GSPE treatment on the intestinal barrier integrity of small and large intestines in diet-induced obese rats. TEER (transepithelial electric resistance) values were estimated from fresh duodenal, ileal and colonic mucosal preparations mounted in an Ussing chamber system. Values represent mean ± SEM; *n* = 8–10. Different letters indicate statistically significant differences between groups (*p* < 0.05); ANOVA test followed by Tukey’s HSD test when variances were equal and Dunnett’s T3 test when they were not.

Barrier integrity in small and large intestine sections was evaluated in an Ussing chamber system by TEER measurements of mucosal preparations from all experimental groups. Similar TEER values were found throughout the intestine of the STD group (24.9 ± 1.8, 25.0 ± 1.3 and 29.4 ± 2.3 ohm.cm^2^ for duodenum, ileum and colon respectively; Figure 3). TEER values in all the intestinal sections from animals fed the CAF diet were significantly lower than those from the STD group (55-70% TEER reduction). Notably, TEER values from the GSPE-treated groups showed a tendency to increase in the duodenum (approx. 54%; Figure 3A) but exhibited statistically significant increases in the ileum (approx. 107%; Figure 3B) and colon (approx. 180%; Figure 3C). Non-statistically significant differences in TEER values were found between the CORR100 and CORR500 groups.

### 3.5. The Expression of Tight Juntion Protein Genes

**Figure 4:**
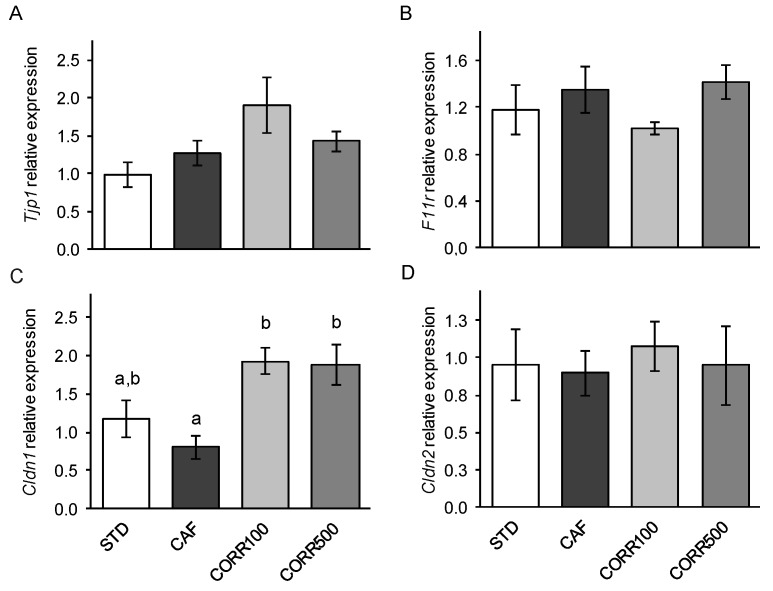
The effect of the GSPE treatment on tight juntion-related gene expression in the ileum of diet-induced obese rats. The expression of target genes was normalized to cyclophilin A gene expression (*Ppia*). Values represent mean ± SEM; *n* = 8–10. Different letters indicate statistically significant differences between groups (*p* < 0.05); ANOVA test followed by Tukey’s HSD test.

To evaluate the influence of GSPE-supplementation on intestinal tight-junction integrity, we analyzed the expression of the genes encoding tight junction-associated proteins in the ileum. The CORR100 and CORR500 groups showed a two-fold increase in the expression of the claudin 1 gene in comparison to the CAF group, without a dose-dependent effect (Figure 4C). The expression of the zonula occludens 1 (Figure 4A), junctional adhesion molecule 1 (Figure 4B) and claudin 2 (Figure 4D) genes did not differ significantly between experimental groups.

### 3.6. Ex Vivo Intestinal Inflammation

**Figure 5:**
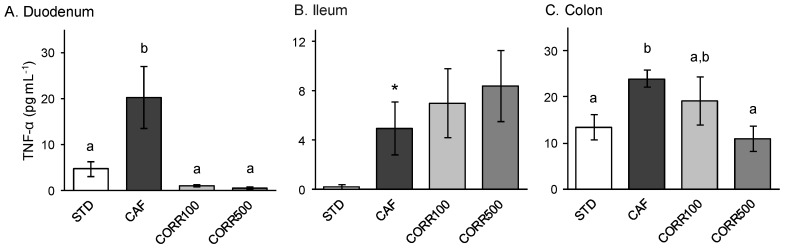
The effect of the GSPE treatment on the local inflammation of small and large intestines in diet-induced obese rats. TNF-α levels were determined from the basolateral media from the Ussing chamber after 30 min of incubation. Values represent mean ± SEM; *n* = 8–10. Different letters indicate statistically significant differences between groups (*p* < 0.05); ANOVA test followed by Tukey’s HSD test when variances were equal and Dunnett’s T3 test when they were not. *Significant statistical differences with respect to the STD group; two-sided Student’s *t*-test.

To evaluate intestinal local inflammation, we determined the level of TNF-α secreted by the intestinal mucosal preparations subjected to Ussing chamber assays in the basolateral media. TNF-α secreted from intestinal tissues in STD diet-fed animals varied between 0.2–13.4 pg mL^−1^, with the highest levels released by the colon (Figure 5). The CAF diet promotes a significant increase in TNF-α secretion from all intestinal tissues in comparison to the STD group (5–20.2 pg mL^−1^), with the ileum being the intestinal section most susceptible to CAF intervention with a 25-fold increase in this marker (Figure 5B). The GSPE-treated group presented lower TNF-α secretions from the duodenum and colon compared to the CAF group. Only the CORR500 treatment was able to reduce the TNF-α secretion to basal levels in the colon (Figure 5C), although both treatments were equally effective in the duodenum (Figure 5A). No GSPE-treatment was effective in reducing the TNF-α secretion from the ileum.

### 3.7. Ileal MPO Activity

MPO activity was determined on ileum tissues as a marker of neutrophil accumulation. The CAF diet induced a 4-fold increase in MPO activity (7.52 and 2.0 U mg^−1^ for the CAF and STD groups respectively). Furthermore, the GSPE treatment reduced MPO activity to levels comparable to the control STD (3.04 and 3.35 U mg^−1^ for the CORR100 and CORR500 groups respectively) without a dose-dependent effect.

### 3.8. Association between Metabolic Endotoxemia and Intestinal Barrier Integrity

**Figure 6:**
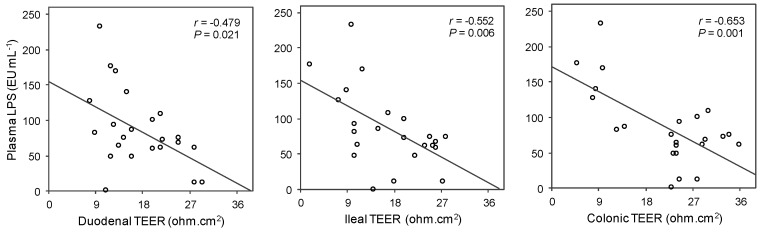
The linear relationship between endotoxemia and TEER values in small and large intestines of rats from the STD, CAF, CORR100 and CORR500 experimental groups. The inset shows the Pearson’s *r* correlation and the corresponding *p*-value; *n* = 23. *p* < 0.05 was considered statistically significant.

To check the association between endotoxemia and integrity of the intestinal barrier we performed Pearson correlation tests between plasma LPS and TEER values determined ex vivo for each intestinal section (Figure 6). We found negative and statistically significant associations for these variables in all the intestinal sections studied. As shown in Figure 6, the strength of the linear relationship increased in the distal intestine.

## 4. Discussion

The intestinal barrier plays a major role in protecting the organism against foreign agents. Its function can be compromised by chronic exposure to diet-related components such as saturated fats and refined carbohydrates [10]. These alterations promote the influx of bacterial endotoxins (i.e., LPS) and exacerbate the immune response. The subsequent systemic inflammation has been associated with obesity-related pathologies [11,13].

Phytochemicals such as flavonoids have been shown to reduce intestinal alterations induced by high saturated-fat/high refined-carbohydrate diets. In this regard, we have found that proanthocyanidins are able to attenuate or even prevent diet-induced alterations to the intestinal barrier and local inflammation [18]. We hypothesized that pharmacological doses of GSPE (100 and 500 mg kg^−1^ bw) may reverse some of the detrimental effects of a long-term obesogenic diet, ameliorating intestinal dysfunction.

We designed an experiment in which diet-induced obese rats received pharmacological doses of GSPE daily for two weeks. Initially, the animals were fed a typical CAF diet, a highly palatable and hypercaloric diet that resembles the Western human diet [29]. The CAF diet is considered a robust inductor of obesity because it is rich in refined carbohydrates as well as saturated fats. In this study, the CAF diet-induced voluntary hyperphagia, resulting in the rapid weight gain and an increased adiposity index compared to animals fed the control STD diet. Similar results have previously been reported in this animal model [3,18,19].

To assess the intestinal barrier permeability and local inflammation we analyzed multiple markers at the end of the experiment. Our results showed lower TEER values in both the small and large intestines and higher levels of plasma OVA in CAF-fed rats after 17 weeks, suggesting a decrease in intestinal epithelial integrity and enhanced paracellular transport. Interestingly, GSPE supplemented animals evidenced the restitution of the intestinal epithelium’s integrity, particularly in the ileum and colon. We also found a significant reduction of the paracellular transportation as a result of CORR100 administration and a tendency toward the reduction in response to CORR500. Thus, GSPE-treatments exerted a clear beneficial effect in the permeability of distal sections of the intestine. Accordingly, a favorable effect of GSPE on the intestinal barrier function was found in a study performed with IL-10-deficient mice that develop spontaneous colitis [30]. In this study, the supplementation with GSPE 0.1% (w v^−1^) in drinking water for 10 weeks reduced significantly in vivo permeation of fluorescently-labeled dextran to the blood after an oral load.

Ex vivo assays also showed that intestinal tissues from animals fed the CAF diet released higher levels of TNF-α. This proinflammatory cytokine is a key mediator of intestinal inflammation that induces TJ opening via myosin light-chain kinase phosphorylation to enhance paracellular flux [31]. Clinical and experimental studies have demonstrated that defects in the intestinal TJs and increased permeability are present in various intestinal and systemic diseases [32]. In the present work, both GSPE-treatments exerted an anti-inflammatory effect on duodenal tissues, reducing TNF-α secretions to basal levels. Nevertheless, only in colon tissues, CORR500 was able to reverse proinflammatory cytokine production. Previous studies have revealed the critical role of the ileum in the pathogenesis of chronic intestinal diseases associated with intestinal permeability alterations and local inflammation [33]. GSPE supplementation did not significantly alter TNF-α secretion from the ileum, but it did promote a decrease in CAF diet-induced neutrophil infiltration as indicated by the reduction in MPO activity. MPO is a key component of oxygen-dependent microbial activity by neutrophils, but it has also been linked to tissue damage in acute or chronic inflammation [34]. In agreement with these findings, a study performed on a rat model of spontaneous inflammatory bowel disease (HLA-B27) found that diet supplementation with 7.6% (w w^−1^) of lyophilized apples rich in flavan-3-ol monomers and proanthocyanidins (2.8 and 26.1 mg g^−1^ respectively), reduced MPO activity along with cyclooxygenase-2 and inducible nitric oxide synthase gene expression in intestinal tissues [35].

Regarding inflammatory parameters, plasma LPS were markedly elevated in the CAF-group at the end of the experiment. Under normal conditions, the presence of LPS in the intestinal lumen does not cause adverse health effects. Nevertheless, the loss of intestinal barrier integrity associated with the consumption of saturated fat/high-sugar diets may favor LPS penetration through the intestinal epithelium to the blood [11]. Two- to three-fold increases in levels of LPS in plasma trigger metabolic endotoxemia, and this process is closely linked to systemic inflammation [12,13]. In the present study, a clear linear relationship between CAF diet-induced TEER loss and LPS levels in plasma was found. This association was stronger in the ileum and colon, suggesting a higher contribution of the distal intestine to metabolic endotoxemia. This observation can be explained by the abundant bacterial population present in distal sections of the intestinal tract [36]. Once it has entered the circulation, LPS activates a series of pro-inflammatory receptors, i.e., toll-like receptors 2 and 4 and the CD14 receptor, leading to cytokine release [13,37]. Accordingly, in this study, the CAF-diet-fed rats also showed higher levels of TNF-α in plasma, indicating a state of systemic inflammation in this group of animals. Remarkably, both GSPE doses were also effective in decreasing plasma LPS to basal levels and tended to reduce the TNF-α release to circulation. These findings are consistent with the observations discussed above regarding GSPE effects on intestinal permeability.

The mechanisms underlying the anti-inflammatory and barrier-protective effects of flavonoids on the intestine were reviewed by Gil-Cardoso et al. [2]. Flavonoids, including proanthocyanidins, reduce intestinal inflammatory processes driven by NF-κB activation, a key factor in pro-inflammatory gene expression. In vitro studies have demonstrated that procyanidin-rich red wine extract down-regulates IκB kinase complex signal transduction, inhibiting the IκB degradation and NF-κB translocation to the nucleus [38]. Another interesting point to consider is the modulation of TJ protein genes by flavonoids. In a recent study, it was observed that Granny Smith apple procyanidin extract increases the expression of TJ protein genes, including occludin and zonula occludens 1 in LPS-treated Caco-2 cells [39]. We demonstrated that CAF diet-induced obese rats supplemented with GSPE (5–500 mg kg^−1^ d^−1^), show an increased expression of zonula occludens 1 [19] and claudin 1 [18] genes in the ileum. A significant increase of claudin 1 protein content has also been estimated in ileal tissues of IL-10 deficient mice supplemented with GSPE 1% (w w^−1^) for 16 weeks [40]. Claudin proteins are considered key components and the structural backbone of TJs [41], with claudin 1 being involved in TJ tightening. Thus, the upregulation of claudin 1 gene expression found in the present work might partially explain the restitution of TEER and intestinal permeability at the ileum level in GSPE-supplemented animals.

Most studies in rodents describing a beneficial effect of polyphenol-rich extracts on diet-induced intestinal dysfunction and metabolic endotoxemia involve preventive or long-term treatment approaches [18,42,43,44]. To our knowledge, there is no other study in the literature describing an ameliorative effect of a polyphenol-rich extract in these alterations as a result of short-term chronic treatment. Data show that pharmacological doses of grape-seed proanthocyanins can normalize intestinal permeability and local inflammation markers altered by a long-term obesogenic diet, as well as the associated metabolic endotoxemia. The effects of both pharmacological doses of GSPE on CAF-diet-induced intestinal dysfunction were similar and, therefore, we found no dose-response relationship. Both doses proved to ameliorate intestinal alterations, but the 100 mg kg^−1^ bw dose seems to be the most effective in correcting intestinal permeability, whereas the 500 mg kg^−1^ bw dose corrected local inflammation more effectively.

GSPE doses used in this study are high but feasible in a translational perspective. We applied a rat-to-human correction factor that relates the body weight and body surface to estimate the theoretical human equivalent doses (HEB) [45]. Considering the 100 mg kg^−1^ bw dose of GSPE, the estimated HED is approximately 0.97 g d^−1^ for a 60 kg person. A clinical study was carried out with 31 volunteers with different body mass index to determine the effect of GSPE administration in postprandial concentrations of plasma LPS after a high-fat breakfast (63 g of fat and 990 kcal) [46]. These authors found that GSPE effectively prevented the postprandial increase of plasma LPS after 4 h compared with the placebo. This effect was statistically significant in the obese group. Another recent clinical study assessed the safety and tolerability of GSPE intake in a small number of healthy adults [47]. This study tested the oral administration of the extract up to 2.5 g d^−1^ for a 4-week period, finding a good tolerability without adverse effects on hematological and biochemical parameters.

## 5. Conclusions

In summary, the consumption of a CAF diet leads to an increase in intestinal permeability and metabolic endotoxemia, which together contribute to the systemic inflammation state present in obese animals. We found that the administration of grape-seed proanthocyanidins not only improves the intestinal health in diet-induced obese rats, effectively ameliorating permeability alterations and local inflammation at the end of a long-term obesogenic diet, but also reduces metabolic endotoxemia. Thus, supplementation with proanthocyanidin-rich natural extracts is outlined as a promising nutritional and therapeutic treatment to ameliorate the diet-induced intestinal dysfunction associated with obesity.

## Figures and Tables

**Table 1:** The composition and energy content of diets administered.

	STD	CAF
**Content (g 100 g^−1^ dry matter)**		
Available carbohydrate	48.0	62.6
Sugar	≈0.0	46.0
Protein	14.3	15.1
Fat	4.0	17.2
Saturated	0.6	8.1
Fiber	4.1	1.7
**Energy contribution**		
kJ g^−1^ dry matter	12.1	20.7
Carbohydrate (%)	67.2	52.0
Protein (%)	20.2	14.1
Fat (%)	12.6	33.9

STD diet, standard chow diet; CAF diet, cafeteria diet. The CAF diet consisted of bacon, sausages, biscuits with paté, carrots, muffins, and sugared milk, which induces voluntary hyperphagia. ≈, approximately.

**Table 2:** The morphometric and biochemical parameters in experimental groups at 17 weeks.

	STD	CAF	CORR100	CORR500
**Morphometric parameters**				
Initial body weight (g)	220.7 ± 4.5	216.6 ± 3.5	221.4 ± 5.3	219.6 ± 3.3
Final body weight (g)	273.7 ± 7.8 ^a^	346.2 ± 12.0 ^b^	358.7 ± 13.3 ^b^	332.7 ± 14.0 ^b^
Body weight gain (g)	48.4 ± 3.4 ^a^	131.3 ± 12.2 ^c^	106.7 ± 12.4 ^b,c^	89.6 ± 6.7 ^a,b^
Adiposity (%)	5.6 ± 0.5	11.8 ± 0.8 ^b^	12.0 ± 1.0 ^b^	11.3 ± 4.9 ^b^
**Biochemical parameters**				
Glucose (mM)	8.9 ± 0.7	10.2 ± 0.1	11.5 ± 1.0	10.4 ± 0.4
Triacylglycerols (mM)	0.41 ± 0.1	0.57 ± 0.1	0.45 ± 0.1	0.46 ± 0.1

STD group, rats fed standard chow diet; CAF group, rats fed cafeteria (CAF) diet; CORR100 group, CAF-fed rats supplemented with a 100 mg kg^−1^ body weight of grape-seed proanthocyanidin extract (GSPE); CORR500 group, CAF-fed rats supplemented with a 500 mg kg^−1^ bw of GSPE. The number of animals included in this study was *n* = 10 for each group. Values indicate mean ± standard error (SEM). Different letters indicate statistically significant differences between groups (*p* < 0.05); ANOVA test followed by Tukey’s HSD test.
